# Prognosis and adjuvant chemotherapy for patients with positive peritoneal cytology in stage IA endometrial cancer

**DOI:** 10.1038/s41598-021-03975-5

**Published:** 2022-01-07

**Authors:** Motoko Kanno, Mayu Yunokawa, Makoto Nakabayashi, Makiko Omi, Ai Ikki, Megumi Mizusaki, Mai Nishimura, Yusuke Shimizu, Kota Okamoto, Yuji Tanaka, Atsushi Fusegi, Sachiho Netsu, Tomoko Kurita, Yoichi Aoki, Terumi Tanigawa, Maki Matoda, Sanshiro Okamoto, Hidetaka Nomura, Kohei Omatsu, Yuko Sugiyama, Kuniko Utsugi, Nobuhiro Takeshima, Hiroyuki Kanao

**Affiliations:** 1 Department of Gynecology, The Cancer Institute Hospital of JFCR, 3-8-31 Ariake, Koutou-ku, Tokyo, 135-8550 Japan; 2 Department of Medical Oncology, The Cancer Institute Hospital of JFCR, Tokyo, Japan

**Keywords:** Medical research, Oncology

## Abstract

This study evaluated the influence of positive peritoneal cytology (PPC) on the prognosis of patients with stage IA endometrial cancer, and the usefulness of adjuvant chemotherapy in their treatment. We retrospectively analyzed the data of patients with stage IA endometrial cancer admitted in our hospital between 2005 and 2015. Among 989 patients who underwent peritoneal cytology, 135 (13.7%) had PPC. Multivariate analysis extracted several independent risk factors for recurrence in stage IA patients, including those with PPC. Adjuvant chemotherapy did not cause a significant difference in the 5-year relapse-free survival rate in patients with PPC (p = 0.78). Similarly, the 5-year recurrence-free survival rate with or without chemotherapy was not different among type II cancer patients (p = 0.11). However, the baseline risk of 5-year relapse-free survival without chemotherapy in patients with PPC and type II was very low (66.7%). While PPC was an independent risk factor for recurrence in stage IA endometrial cancer, adjuvant chemotherapy did not influence the survival rate in patients with PPC. While it is controversial whether adjuvant chemotherapy should be administered in stage IA uterine cancer with only PPC as a prognostic factor, it should be considered for early-stage patients who have multiple risk factors for recurrence.

## Introduction

Endometrial cancer is the most common gynecological cancer in advanced countries, with 400,000 cases per year worldwide and 60,000 cases per year in the United States; its prevalence is expected to further increase with time^1,2^. Most patients are diagnosed in the early stage because symptoms of atypical genital bleeding develop in more than 90% of patients. When reported early, approximately 75 to 80% of endometrial cancer patients are diagnosed at Federation of Gynecology and Obstetrics (FIGO) Stage I, and stage IA patients are known to have a favorable prognosis with a 5-year survival rate ≥ 90%^3,4^.

Positive peritoneal cytology (PPC) has been reported to be a poor prognostic factor for endometrial cancers, but it is not included the International FIGO 2009 stage classification. Studies show that PPC does not influence the prognosis of stage III cancers unlike other factors like lymph node metastasis, and uterine serosal or adnexal metastasis, but it is unclear whether PPC influences the prognosis in patients with early stage endometrial cancer (EEC)^5–7^. Although some studies have reported PPC to be a risk factor for recurrence in patients with EEC, other studies have not found PPC to be an independent risk factor for occurrence. So, no consensus has been established^8–11^.

Several guidelines recommend postoperative adjuvant therapy for EEC patients having risk factors for recurrence^3,4^. However, the efficacy of adjuvant therapy for patients with stage IA endometrial cancer is unclear, because most clinical trials that evaluated the efficacy of adjuvant therapy included stage I patients with deep myometrial invasion, but almost excluded stage IA with myometrial invasion within 50%^12–14^.

The objective of this study was to evaluate the influence of potential independent risk factors like PPC on the prognosis of patients with very early stage, stage IA endometrial cancer, and to assess the usefulness of postoperative adjuvant chemotherapy in its treatment.

## Material and methods

### Patient population

This was a retrospective comparative study involving data on patients with endometrial cancer (FIGO stage IA) who received their first treatment at The Cancer Institute Hospital of JFCR between January 2005 and December 2015. The study was approved by The Cancer Institute Hospital of JFCR Review Board and was conducted in accordance with the relevant guidelines and regulations of the Institutional Review Board. We obtained signed informed consent from participants. The primary endometrial cancer patients (FIGO stage IA) who underwent standard surgery were eligible for this study. We excluded the following patients: those who opted for non-surgical treatment, including uterine preservation treatment for fertility preservation and radiation therapy; those who died from another disease; those who had undergone a hysteroscopic examination before surgery; patients who received preoperative hormone therapy or chemotherapy; and patients who had multiple cancers (thus affecting the EEC treatment protocol)^15,16^.

### Study definition

Surgical staging was performed using the FIGO 2009 staging system^17^. According to this classification, an invasion of < 50% of the uterine muscle is classified as stage IA. Concerning the tumor grading, the World Health Organization (WHO) 2014 classification was used^18^. Ages and body mass index (BMI) were each grouped into two categories (ages < 60 and ages ≥ 60; BMI < 25 and BMI ≥ 25 kg/m^2^, respectively) on the basis of the grouping done in previous studies^19,20^. A False PPC was classified as negative according to Japanese guidelines^4^. The relapse-free survival time was defined as the time lapse between the first recurrence and the day of the surgery or (in patients without recurrence) the time lapse between the day of the surgery and last consultation (follow-up) day. The overall survival time was defined as the time lapse between the day of the surgery and the date of death or the last consultation day (for patients who were still alive). The patterns of recurrence included locoregional recurrences (defined as vaginal or intrapelvic recurrences) and distant recurrences (upper para-aortic lymph node metastasis, peritoneal dissemination, and metastasis to other organs).

### Surgery and adjuvant chemotherapy

We usually evaluate tissue diagnosis by endometrial biopsy or curettage, myometrial invasion by dynamic MRI, and lymph node and distant metastasis by CT scan or PET/CT to determine the preoperative clinical stage and surgical procedure. Hysteroscopic biopsy was used when endometrial tissue biopsy or curettage failed to make a diagnosis. All laparotomies were attended by a qualified Japanese gynecologic oncologist as a surgeon or first assistant. In addition, Japanese gynecological oncologists and laparoscopic technology certified doctors participated in all laparoscopic surgery as surgeons or first assistants. The basic surgical procedures used to treat EEC in our hospital included: total hysterectomy, bilateral salpingo-oophorectomy, and regional lymph node biopsy/dissection. All patients underwent hysterectomy, and two types of hysterectomy procedures could be performed: simple total hysterectomy and modified radical hysterectomy. Omentectomy was performed in patients with non-endometrioid adenocarcinoma, or with PPC. Regional lymph node dissection was omitted (in some patients) when type I lymph node enlargement and uterine muscle invasion were absent on imaging diagnosis. Patients in whom a lymph node biopsy was performed without systematic dissection were grouped with patients without dissection. The choice between laparotomy and laparoscopy was made based on the size of the complicating uterine fibroids. Specimens for peritoneal fluid cytology were collected from the Douglas pouch by aspiration or washing at the initiation of surgery.

Adjuvant chemotherapy was recommended if the patient had any one of the following risk factors: Type II EEC, vascular invasion, myometrial invasion within 50%, and PPC in patients with FIGO stage I^4^. Six courses of chemotherapy were administered, and the regimens used were:

- AP (adriamycin 60/m^2^, cisplatin 50/m^2^) (day 1, q21days),
- DP (docetaxel 70/m^2^, cisplatin 60/m^2^) (day 1, q21days), and
- TC (paclitaxel 175/m^2^, carboplatin AUC = 6) (day 1, q21days).

Patients who received three or more courses were included for analysis in this study^21^.

### Statistical analysis

Characteristics were compared using Fisher’s exact test. For the analysis of the period of recurrence, Cox’s regression analysis was used, and univariate and multivariate analyses were performed to identify the prognostic factors. The relapse-free survival curve and overall survival curve were prepared using the Kaplan–Meier method. P-values < 0.05 were considered statistically significant. The hazard ratio and 95% confidence interval were calculated. The software Easy R (EZR) Version 1.38 was used for statistical analyses^22^.

### Ethical approval and consent for participation and consent for publication

We applied Opt-out method to obtain consent on this study by using our hospital home page. We conducted this study in accordance with the Declaration of Helsinki, and the protocol was accepted by the Ethics Committee of the Cancer Institute Ariake Hospital, JFCR (approval number, No 2018-1238).

## Results

### Patients’ characteristics

**Figure. 1:**
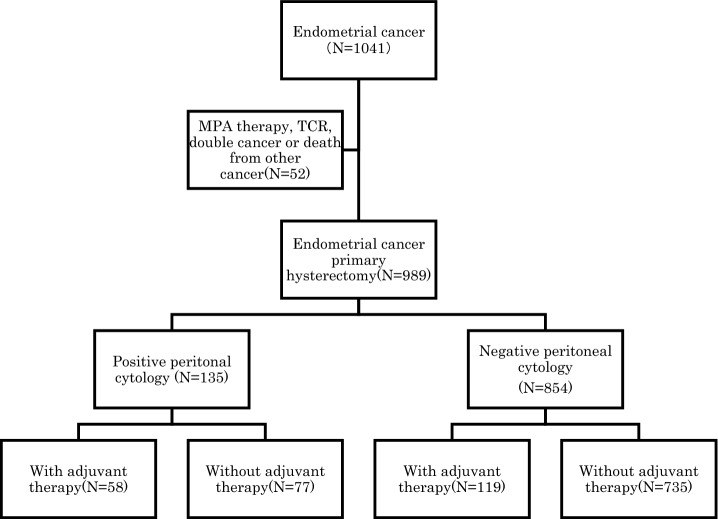
Flow chart of patient recruitment.

In total, 1041 patients with stage IA endometrial cancer were admitted into our institution during the study period and 989 were eligible for inclusion in this study (Fig. 1). The median age was 55 years (range 26–87 years). The median duration of follow-up for all the patients was 70 months (range 2–166 months). Out of the 989 patients who underwent peritoneal cytology, 135 patients (13.7%) were positive (Fig. 1) (Table 1). The 5-year relapse-free survival rate and 5-year survival rate of 989 patients with stage IA were 95.6% and 98.3%, respectively.

**Table 1:** Patient, disease, and treatment characteristics of all the patients (n = 989).

Variable	N = 989	(%)
**Age years**		
Median (years)	55	
Range	26–87	
BMI < 25	717	72.5
BMI ≥ 25	272	27.5
**Histological type**		
Type I	820	82
Endometrioid G1	573	63
Endometrioid G2	234	26
Mucinous	3	0.3
Mixed (G1, G2, mucinous)	10	1.1
Type II	169	18
Endometrioid G3	52	5.7
Serous	22	2.4
Clear	12	1.3
Carcinosarcoma	34	3.7
Mixed (type II)	49	5.4
**Peritoneal cytology**		
Positive	135	14
Negative	854	86
With myometrial invasion	556	56.2
Without myometrial invasion	433	43.8
LSVI positive	104	89.5
LSVI negatige	885	10.5

*BMI* body mass index; Without myometrial invasion: endometrial cancer in which the malignant cells are histopathologically found only in the endometrium; myometrial invasion: endometrial cancer with myometrial invasion within 50%; *LSVI* lymphovascular space invasion.

### Univariate and multivariate analysis for recurrence

Univariate analysis was performed to identify the risk factors for recurrence (Table 2). In univariate analysis, the poor prognostic factors identified were age ≥ 60 years, BMI ≥ 25 kg/m^2^, type II cancer, PPC, muscle invasion, and lymphovascular space invasion. In multivariate analysis, BMI ≥ 25 kg/m^2^, type II cancer, muscle invasion, and PPC were extracted as independent risk factors for recurrence (Supplementary Fig. 1A–F) (Table 2). The 5-year relapse-free survival rates were significantly lower in patients with PCC (96.2% vs. 91.8%, p = 0.002) (Supplementary Fig. 2).

**Table 2:** Risk factors for recurrence of endometrial carcinoma stage IA (univariate and multivariate analysis).

Characteristic	N	Univariate HR (95% CI)	P-value	Multivariate HR (95% CI)	P-value
**Age**					
< 60	677	1	**0.012**	1	0.085
≥ 60	312	2.08(1.73–3.70)		1.69(0.93–3.06)	
**BMI**					
< 25	717	1	**0.017**	1	**0.002**
≥ 25	272	2.03(1.13–3.66)		2.52(1.40–4.54)	
**Histological type**					
Type I	820	1	** < 0.001**	1	** < 0.001**
Type II	169	3.83(2.14–6.81)		3.24	
**Peritoneal cytology**					
Negative	854	1	**0.003**	1	**0.024**
Positive	135	2.58(1.36–4.89)		2.152(1.11–4.18)	
**Myometrial invasion**					
Without myometrial invasion	433	1	**0.005**	1	**0.049**
With myometrial invasion	556	2.62(1.33–5.16)		2.03(1.00–4.09)	
**LVSI**					
Negative	885	1	**0.004**	1	0.39
Positive	104	2.65(1.35–5.21)		1.38(0.66–2.89)	

*BMI* body mass index, *LVSI* lymphovascular space invasion, *CI* confidence interval, *HR* Hazard ratio.

To clarify the risk factors for recurrence in patients with PPC stage IA, we performed the univariate and multivariate analysis in 135 patients with PPC stage IA endometrial cancer. In univariate analysis of stage IA EEC patients with PPC, the risk factors for recurrence were age ≥ 60 years and type II cancer. In multivariate analysis, type II cancer was identified as an independent risk factor (Table 3).

**Table 3:** Risk factors for recurrence in peritoneal cytology-positive, endometrial carcinoma stage IA patients (univariate and multivariate analysis).

	N	HR (95% CI)	P-value	HR (95% CI)	P-value
**Age (range)**					
< 60	107	1	**0.014**	1	0.088
≥ 60	28	3.96 (1.31–11.9)		2.86 (0.85–9.56)	
**BMI**					
< 25	100	1	0.160		
≥ 25	35	2.28 (0.71–7.20)			
**Histology**					
Type I	99	1	**0.006**	1	**0.035**
Type II	36	4.76 (1.55–14.5)		3.56 (1.08–11.6)	
**Tumor size**					
≥ 3 cm	78	1	0.153		
< 3 cm	57	0.44 (0.14–1.35)			
**Surgery**					
Laparoscopy	17	1	0.152		
Laparotomy	118	0.38 (0.10–1.43)			
**Myometrial invasion**					
Without myometrial invasion	43	1	0.54		
With myometrial invasion	92	0.71 (0.23–2.16)			
**LVSI**					
present	27	1	0.669		
absent	108	0.71 (0.16–3.25)			
**Lymphadenectomy**					
Performed	92	1	0.965		
No performed	43	0.96 (0.29–3.18)			
With Adj chemotherapy	58	1	0.781		
Without Adj chemotherapy	77	1.17 (0.39–3.48)			

*BMI* body mass index, *LVSI* lymphovascular space invasion, *Adj* Adjuvant.

The 5-year relapse-free survival rates were significantly lower in type II patients with stage IA EEC with PPC (97% vs. 78.6%, p < 0.001) (Supplementary Fig. 3).

### The efficacy of postoperative adjuvant chemotherapy

In 135 patients with PPC, 13 (9.6%) developed a recurrence. Out of the 58 patients who received adjuvant chemotherapy, 6 (10.3%) developed recurrences (4 patients had distant metastases and 2 had local recurrences). Out of the 77 patients who did not receive adjuvant chemotherapy, 7 patients (9.1%) developed recurrences (1 had a local recurrence and 6 had distant metastases).

**Figure 2:**
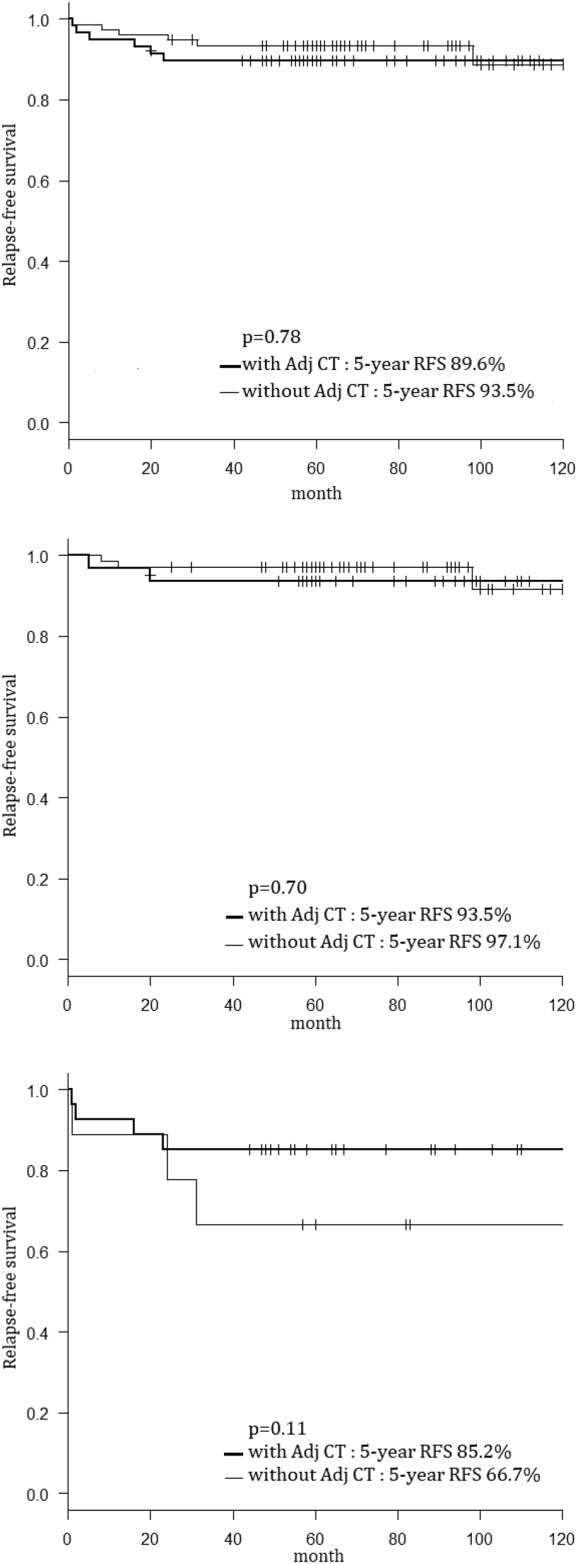
1. Kaplan–Meier curves for the relapse-free survival rates of PPC stage IA endometrial cancer patients with and without adjuvant chemotherapy. *PPC* positive peritoneal cytology, *Adj CT* Adjuvant chemotherapy, *RFS* Relapse-free survival. 2. Kaplan–Meier curves for the relapse-free survival rates of PPC stage IA endometrial cancer patients with and without adjuvant chemotherapy (Type I). *PPC* positive peritoneal cytology, *RFS* Relapse-free survival. 3. Kaplan–Meier curves for relapse-free survival rates of PPC stage IA endometrial cancer patients with and without adjuvant chemotherapy (type II). *PPC* positive peritoneal cytology, *RFS* Relapse-free survival.

For the stage IA EEC patients with PPC, there was no significant difference in the 5-year relapse-free survival rate between those who did not receive adjuvant chemotherapy and those who did received (93.5% against 89.6%, p = 0.78) (Fig. 2-1).

The relapse-free survival rates of patients with and without adjuvant chemotherapy were analyzed with the cancer type (type 1, n = 99; type 2, n = 36). In patients with type I cancer, there was no significant difference in the 5-year relapse-free survival rate between patients with and those without adjuvant chemotherapy (93.5% against 97.1%, p = 0.70) (Fig. 2-2). Similarly, the difference between these two groups was not significant in patients with type II (n = 36) (85.2% against 66.7%, p = 0.11) (Fig. 2-3).

Also, in patients with type II cancer, there was no significantly difference in the 5-year overall survival rate between patients with and those without adjuvant chemotherapy (91.9% versus 77.8%, p = 0.15) (Supplementary Fig. 4).

## Discussion

This study found that PPC, alongside a BMI ≥ 25, type II cancer, and muscle invasion, was an independent poor prognostic factor in patients with stage IA EEC. In stage IA EEC with PPC, type II cancer was the only independent poor prognostic factor. The administration of adjuvant chemotherapy was not associated with a longer relapse-free survival in all the stage IA EEC patients with PPC.

Several studies have reported that PPC is a risk factor for cancer recurrence in patients with EEC (stage I and II) after revision of the FIGO 2009 staging^8–11^. In our study which focused on stage IA EEC, PPC was an independent risk factor for recurrence (p = 0.036). Wang et al. however reported that PPC does not influence the overall survival rate of patients with stage IA EEC^23^. Previous reports excluded non-endometrial cancers from the analysis, which may have led to different results from our study.

In fact, our study found no difference in the overall survival between patients with stage IA with or without PPC when statistics were taken for type I histology (data not shown). The European Society for Medical Oncology guidelines recommend the collection of peritoneal cytology information, especially in patients with tumors of non-endometrioid histology. This is because a retrospective study showed that PPC has a prognostic value^3^. In the present study, PPC was a risk factor for recurrence in stage IA endometrial cancer, and it is necessary to carefully manage it.

Atypical cells in peritoneal cytology were considered negative on the basis of the Japanese guidelines^4^. Matsuo et al. suggested that the presence of malignant cells or atypical cells on peritoneal cytology should be defined abnormal peritoneal cytology^24^. Presently, we decided to create a cell block in case of atypical cells. The cell block method prepares paraffin blocks from specimens collected for cytology. After centrifugation, formalin fixation, and paraffinization, paraffin blocks containing tissue specimens of atypical cells allow histological diagnosis of benign or malignant cells. In the future, we need to examine the treatment of atypical cells, including their prognosis.

Matsuo et al. reported that adjuvant chemotherapy for PPC in EEC (stages I and II) may reduce the incidence of distant metastasis^24^. Seagle et al. also reported that adjuvant chemotherapy increased the survival rate of EEC with PPC (stage I and II) patients^25^. However, Wang et al. reported that adjuvant chemotherapy did not improve the outcome of PPC stage IA EEC (with endometrioid carcinoma)^23^. In our study, adjuvant chemotherapy for IA EEC with PPC did not improve the relapse-free survival. The 5-year relapse-free survival rate of stage IA EEC patients with PPC without chemotherapy was 93.8% in the overall population. These results suggest that adjuvant chemotherapy was not always necessary for all stage IA patients with PPC because the baseline risk for recurrence in these patients was very low. We should select patients with higher risk for recurrence among stage IA EEC patients with PPC.

In addition to advanced stage, high-grade endometrial cancer or non-endometrial cancer, defined as type II subtype and the presence of vascular and/or muscle invasion, have been reported as poor prognostic factors^3,4,26^. Melody, Tatebe, and Donovan et al. reported studies on stage IA serous adenocarcinoma and clear cell cancer among type II patients in which postoperative radiotherapy and chemotherapy prolonged the relapse-free survival^27–29^.

Our study has several limitations. Because of the retrospective nature of the study, the necessary clinical factors in endometrial cancer, including the presence of diabetes, socio-economic status, and molecular classification of endometrial cancer, could not be sufficiently obtained. In addition, the data includes only Japanese patients, with some differences in the reports from the West due to racial characteristics. For example, the median BMI was lower than that reported in other studies. We considered that a lower median BMI was related to regional factors. In a previous report from Japan, the BMI was 22.5–24.1, which is similar to the present study^30,31^.

In our study, type II cancer was an independent risk factor for recurrence in stage IA EEC patients with PPC; therefore, we investigated the efficacy of adjuvant chemotherapy as a function to the cancer type. Adjuvant chemotherapy for type I patients (stage IA with PPC) did not improve the relapse-free survival, with a good baseline risk (the 5-year relapse-free survival rate was 93.5%). In addition, adjuvant chemotherapy for type II patients did not significantly improve relapse-free survival. However, the baseline risk of 5-year relapse-free survival without chemotherapy was very low (66.7%). Although there was no significant difference, the 5-year relapse-free survival rate of patients who received adjuvant chemotherapy with PPC stage IA type II EEC was 85.2%. On the other hand, the 5-year relapse-free survival rate of patients who did not receive adjuvant chemotherapy is 66.7%. Therefore, it may be better to treat them with adjuvant chemotherapy. However, the small sample size of patients and relapse events in this study was the most important limitation in evaluating the effect of adjuvant chemotherapy for stage IA patients with PPC. To prove the effect of postoperative treatment in a population with a very good prognosis, a very large sample size is required to statistically detect small differences.

Analyses involving a larger number of patients and prospective trials need to be carried out to ascertain the need for adjuvant chemotherapy in early stage patients with type II cancers. This lack of significant difference in our study could be due to the small sample size we used. More importantly, it is necessary to select treatment taking into consideration the risk for recurrence of individual factors because the baseline risk for recurrence was worse when multiple risk factors were present^32^.

## Conclusion

In patients with stage IA EEC, PPC is an independent risk factor for recurrence. Adjuvant chemotherapy was not recommended for all stage IA EEC patients with PPC. However, by analyzing the cancer type, the presence of a population with a higher risk for recurrence was confirmed. It is therefore necessary to investigate the efficacy of adjuvant chemotherapy in patients with early stage EEC having risk factors for recurrence, especially those with many factors.

## Supplementary Information

Supplementary Information.

## Data Availability

The data that support the findings of this study are available from the corresponding author, [M.Y.], upon reasonable request.

## References

[1] Siegel RL, Miller KD, Jemal A (2019). Cancer statistics, 2019. CA A Cancer J. Clin..

[2] Estimated number of new cases in 2020, worldwide, both sexes, all ages https://gco.iarc.fr/today/online-analysis-table?v=2020&mode=cancer&mode_population=continents&population=900&populations=900&key=asr&sex=0&cancer=39&type=0&statistic=5&prevalence=0&population_group=0&ages_group%5B%5D=0&ages_group%5B%5D=17&group_cancer=1&include_nmsc=1&include_nmsc_other=1

[3] Colombo N, Creutzberg C, Amant F, Bosse T, Gonzalez-Martin A, Ledermann J (2015). ESMO-ESGO-ESTRO consensus conference on endometrial cancer: Diagnosis, treatment and follow-up. Radiother. Oncol..

[4] Ebina Y, Katabuchi H, Mikami M, Nagase S, Yaegashi N, Udagawa Y (2016). Japan Society of Gynecologic Oncology guidelines 2013 for the treatment of uterine body neoplasms. Int. J. Clin. Oncol..

[5] Werner HM, Trovik J, Marcickiewicz J, Tingulstad S, Staff AC, Amant F (2012). Revision of FIGO surgical staging in 2009 for endometrial cancer validates to improve risk stratification. Gynecol. Oncol..

[6] Pecorelli S (2009). Revised FIGO staging for carcinoma of the vulva, cervix, and endometrium. Int. J. Gynaecol. Obstet..

[7] Kim HS, Song YS (2009). International Federation of Gynecology and Obstetrics (FIGO) staging system revised: What should be considered critically for gynecologic cancer?. J. Gynecol. Oncol..

[8] Milgrom SA, Kollmeier MA, Abu-Rustum NR, Makker V, Gardner GJ, Barakat RR (2013). Positive peritoneal cytology is highly predictive of prognosis and relapse patterns in stage III (FIGO 2009) endometrial cancer. Gynecol. Oncol..

[9] Garg G, Gao F, Wright JD, Hagemann AR, Mutch DG, Powell MA (2013). Positive peritoneal cytology is an independent risk-factor in early stage endometrial cancer. Gynecol. Oncol..

[10] Scott SA, van der Zanden C, Cai E, McGahan CE, Kwon JS (2017). Prognostic significance of peritoneal cytology in low-intermediate risk endometrial cancer. Gynecol. Oncol..

[11] de Boer SM, Powell ME, Mileshkin L, Katsaros D, Bessette P, Haie-Meder C (2018). Adjuvant chemoradiotherapy versus radiotherapy alone for women with high-risk endometrial cancer (PORTEC-3): Final results of an international, open-label, multicentre, randomised, phase 3 trial. Lancet Oncol..

[12] Randall ME, Filiaci V, McMeekin DS, von Gruenigen V, Huang H, Yashar CM (2019). Phase III trial: Adjuvant pelvic radiation therapy versus vaginal brachytherapy plus paclitaxel/carboplatin in high-intermediate and high-risk early stage endometrial cancer. J. Clin. Oncol..

[13] Nomura H, Aoki D, Michimae H, Mizuno M, Nakai H, Arai M (2019). Effect of taxane plus platinum regimens vs doxorubicin plus cisplatin as adjuvant chemotherapy for endometrial cancer at a high risk of progression. A randomized clinical trial. JAMA Oncol..

[14] Kasamatsu T, Onda T, Katsumata N, Sawada M, Yamada T, Tsunematsu R (2003). Prognostic significance of positive peritoneal cytology in endometrial carcinoma confined to the uterus. Br. J. Cancer..

[15] Chang YN, Zhang Y, Wang YJ, Wang LP, Duan H (2011). Effect of hysteroscopy on the peritoneal dissemination of endometrial cancer cells: A meta-analysis. Fertil. Steril..

[16] Takac I, Zegura B (2007). Office hysteroscopy and the risk of microscopic extrauterine spread in endometrial cancer. Gynecol. Oncol..

[17] Creasman W (2009). Revised FIGO staging for carcinoma of the endometrium. Int. J. Gynaecol. Obstet..

[18] Kurman, R.J., International Agency for Research on C, World Health O (2014). WHO Classification of Tumours of Female Reproductive Organs.

[19] Creutzberg CL, van Putten WLJ, Koper PCM, Lybeert MLM, Jobsen JJ, Wárlám-Rodenhuis CC (2000). Surgery and postoperative radiotherapy versus surgery alone for patients with stage-1 endometrial carcinoma: multicentre randomised trial. Lancet.

[20] Ward KK, Roncancio AM, Shah NR, Davis MA, Saenz CC, McHale MT (2013). The risk of uterine malignancy is linearly associated with body mass index in a cohort of US women. Am. J. Obstet. Gynecol..

[21] Gao M, Zhang N, Song N, Zheng H, Yan X, Gao Y (2018). Chemotherapy as adjuvant treatment for early stage endometrial cancer with high intermediate risk factors. Int. J. Gynecol. Cancer..

[22] Kanda Y (2013). Investigation of the freely available easy-to-use software 'EZR' for medical statistics. Bone Marrow Transplant..

[23] Wang L, Li L, Wu M, Lang J (2020). The prognostic role of peritoneal cytology in stage IA endometrial endometrioid carcinomas. Curr. Probl. Cancer..

[24] Matsuo K, Yabuno A, Hom MS, Shida M, Kakuda M, Adachi S (2018). Significance of abnormal peritoneal cytology on survival of women with stage I–II endometrioid endometrial cancer. Gynecol. Oncol..

[25] Seagle BL, Alexander AL, Lantsman T, Shahabi S (2018). Prognosis and treatment of positive peritoneal cytology in early endometrial cancer: Matched cohort analyses from the National Cancer Database. Am. J. Obstet. Gynecol..

[26] Morice P, Leary A, Creutzberg C, Abu-Rustum N, Darai E (2016). Endometrial cancer. Lancet.

[27] Qu XM, Velker VM, Leung E, Kwon JS, Elshaikh MA, Kong I (2018). The role of adjuvant therapy in stage IA serous and clear cell uterine cancer: A multi-institutional pooled analysis. Gynecol. Oncol..

[28] Tatebe K, Hasan Y, Son CH (2019). Adjuvant vaginal brachytherapy and chemotherapy versus pelvic radiotherapy in early-stage endometrial cancer: Outcomes by risk factors. Gynecol. Oncol..

[29] Donovan E, Reade CJ, Eiriksson LR, Pond GR, Arora N, Elit L (2018). Outcomes of adjuvant therapy for stage IA serous endometrial cancer. Cureus..

[30] Kawachi A, Shimazu T, Budhathoki S, Sawada N, Yajima T, Iwasaki M (2019). Association of BMI and height with the risk of endometrial cancer, overall and by histological subtype: A population-based prospective cohort study in Japan. Eur. J. Cancer Prev..

[31] Yunokawa M, Sasada S, Takehara Y, Takahashi K, Shimoi T, Yonemori K (2019). Real-world data on initial treatment strategies for older adult patients with endometrial cancer in Japan. Cancer Chemother. Pharmacol..

[32] Takahashi K, Yunokawa M, Sasada S, Takehara Y, Miyasaka N, Kato T (2019). A novel prediction score for predicting the baseline risk of recurrence of stage I–II endometrial carcinoma. J. Gynecol. Oncol..

