# Supplementary material for: Prognosis and adjuvant chemotherapy for patients with positive peritoneal cytology in stage IA endometrial cancer
Source: Sci Rep. 2022 Jan 7;12:166. doi: 10.1038/s41598-021-03975-5 (PMC8741827; doi:10.1038/s41598-021-03975-5)

# Supplemental Materials

# Figure legend

Supplementary Figure 1A. Kaplan–Meier curves for the age-specific relapse-free survival rates of stage IA endometrial cancer patients.

Supplementary Figure 1B. Kaplan–Meier curves for the BMI-specific relapse-free survival rates of stage IA endometrial cancer patients.

Supplementary Figure 1C. Kaplan–Meier curves for the type-specific relapse-free survival rates of stage IA endometrial cancer patients.

Supplementary Figure 1D. Kaplan–Meier curves for the peritoneal cytology-specific relapse-free survival rates of stage IA endometrial cancer patients.

Supplementary Figure 1E. Kaplan–Meier curves for the myometrial invasion- specific (MI) relapse-free survival rates of stage IA endometrial cancer patients.

Supplementary Figure 1F. Kaplan–Meier curves for the lymphovascular space invasion-specific (LVSI) relapse-free survival rates of stage IA endometrial cancer patients.

Supplementary Figure 2. Kaplan–Meier curves for the relapse-free survival rates of PPC and NPC stage IA endometrial cancer patients.

NPC: negative peritoneal cytology; PPC: positive peritoneal cytology; RFS: Relapse-free survival

Supplementary Figure 3. Kaplan–Meier curves for the relapse-free survival rate of PPC stage IA endometrial cancer patients for different histological types.

PPC: positive peritoneal cytology; RFS: Relapse-free survival

Supplementary Figure. 4. Kaplan–Meier curves for overall survival rates of PPC stage IA endometrial cancer patients with and without adjuvant chemotherapy (type II).

PPC: positive peritoneal cytology

# Supplementary Figure 1A


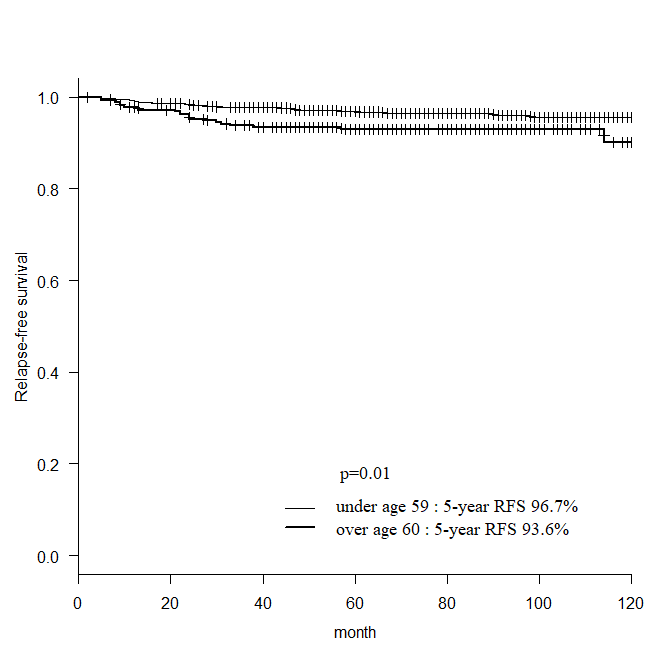


# Supplementary Figure 1B


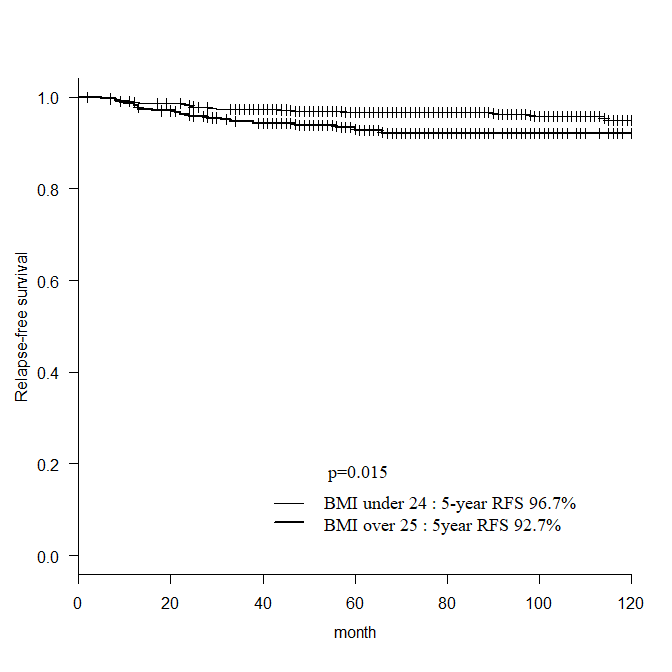


# Supplementary Figure 1C


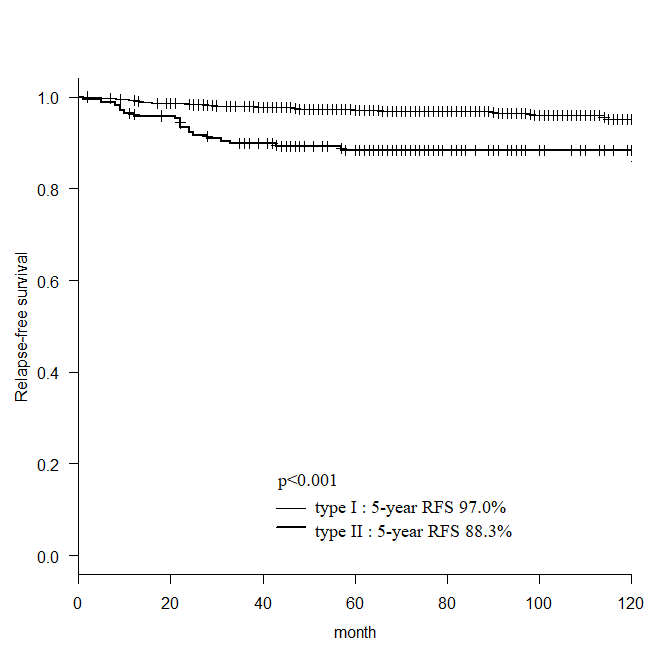


# Supplementary Figure 1D


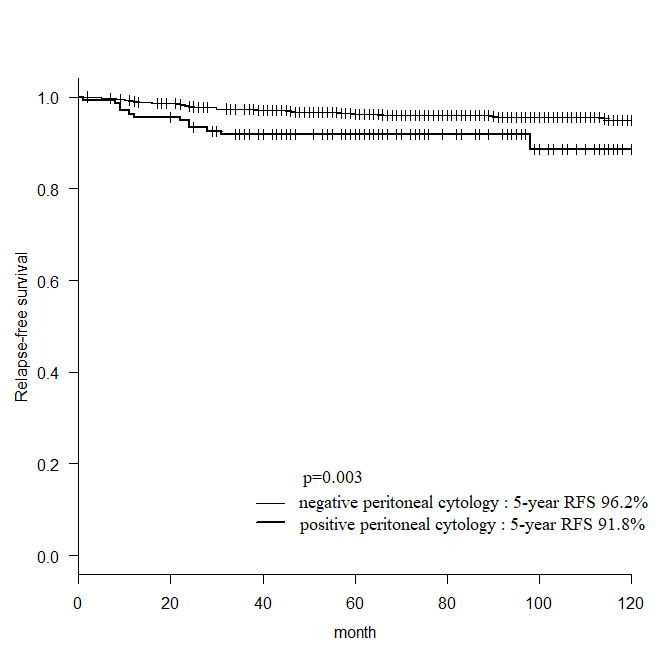


# Supplementary Figure 1E


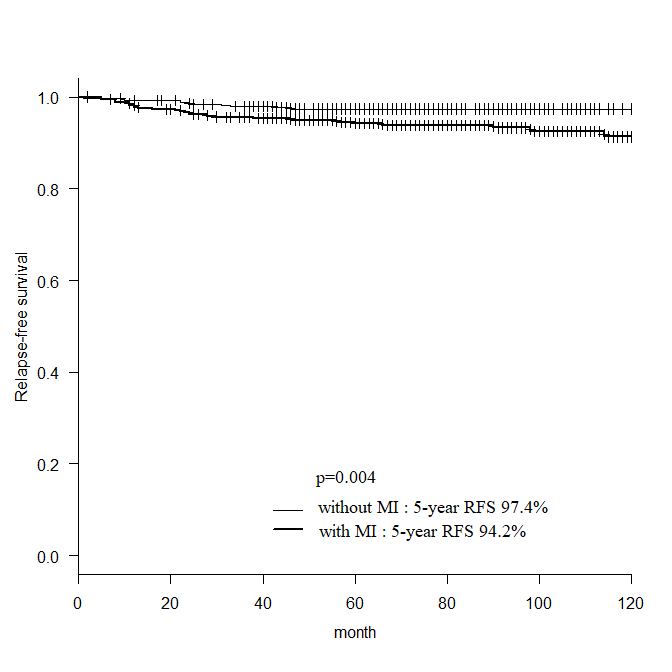


# Supplementary Figure 1F


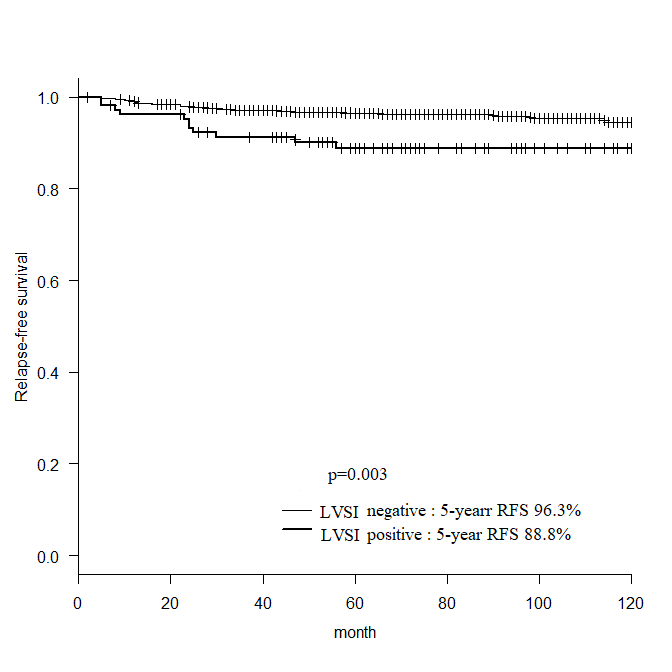


# Supplementary Figure 2


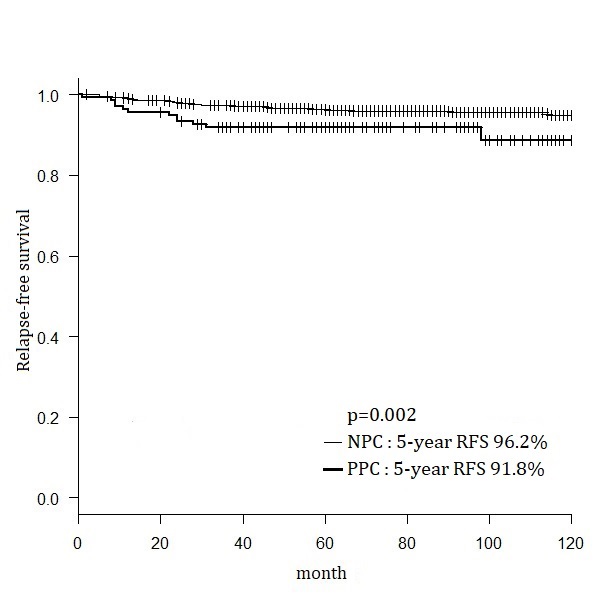


# Supplementary Figure 3


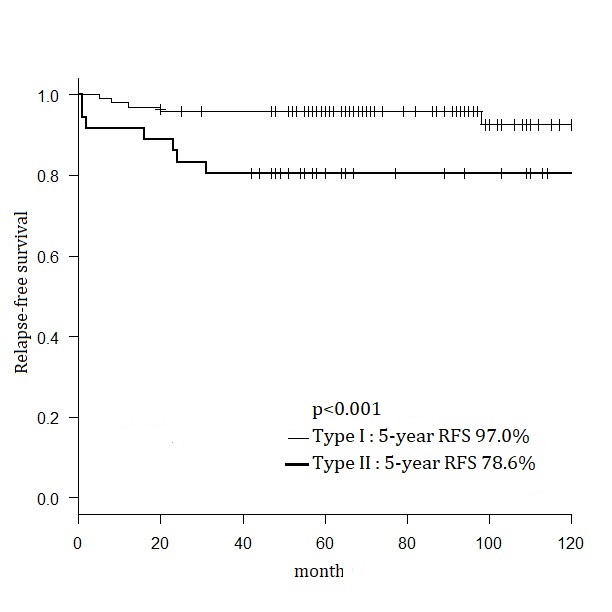


# Supplementary Figure 4


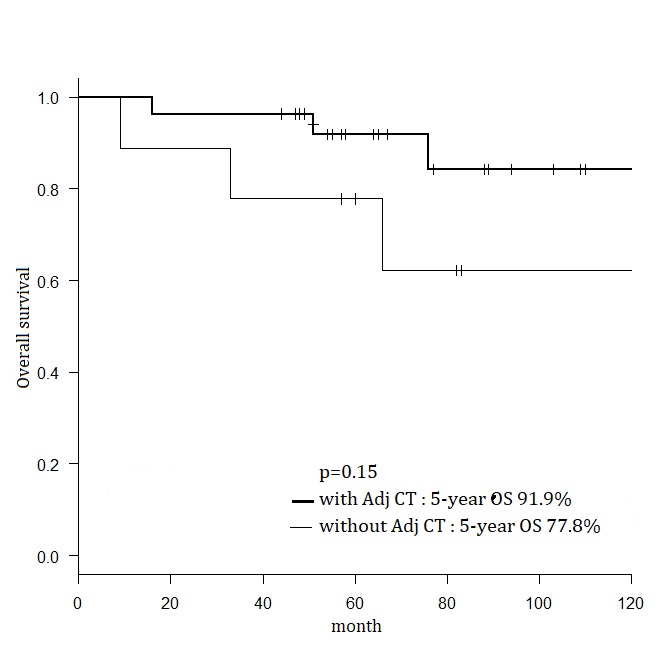

Supplement: Supplementary file 1 — Supplementary Information. [file 41598_2021_3975_MOESM1_ESM.docx]
